# Supplementary material for: Paths and pathways that generate cell-type heterogeneity and developmental progression in hematopoiesis
Source: eLife. 2021 Oct 29;10:e67516. doi: 10.7554/eLife.67516 (PMC8610493; doi:10.7554/eLife.67516)
Supplement: Supplementary file 2. [file elife-67516-supp2.pdf]

Cluster gene lists from Cho et al. mapped onto scRNA-Seq data from this study

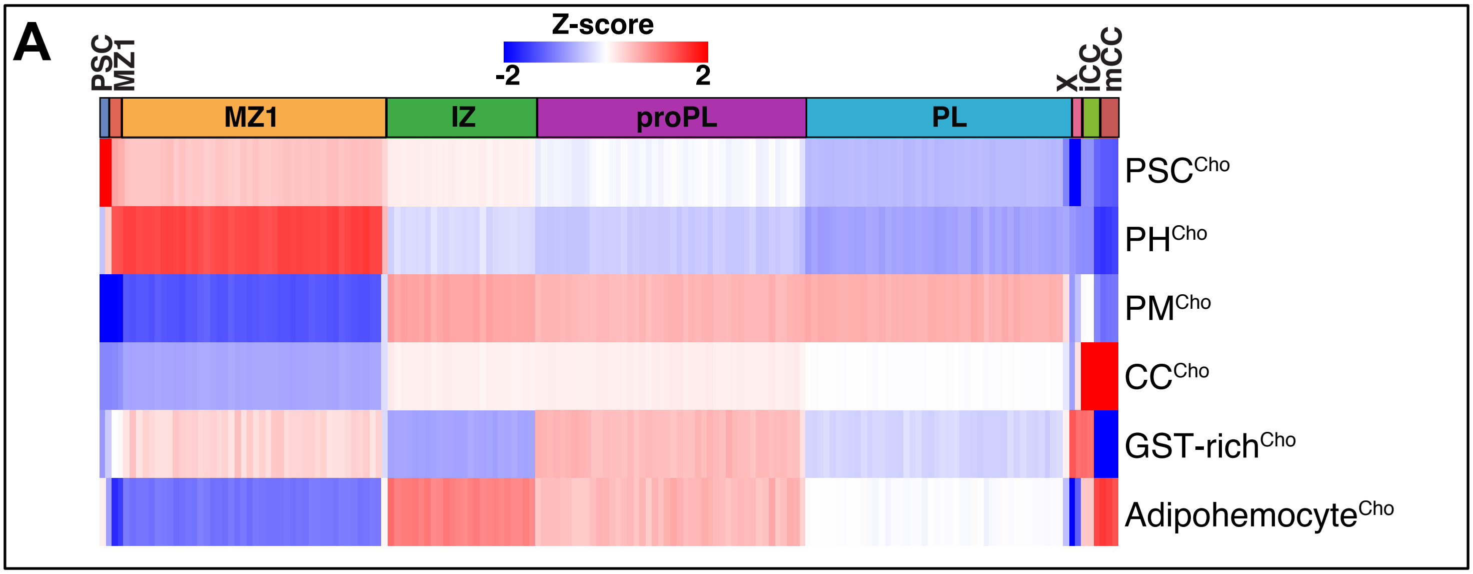

Comparison table of Cluster gene lists from Cho et al. to this study

B highest number of genes in common; second highest number of genes in common

	PSC (368)	MZ1 (187)	MZ2 (97)	IZ (266)	proPL (73)	PL (973)	CC (442)	X (2525)
PSC^Cho (243)	145	13	14	17	4	24	29	20
PH^Cho (72)	10	38	33	2	0	2	4	8
PM^Cho (214)	17	0	1	103	51	153	32	48
CC^Cho (179)	9	4	1	11	5	15	151	13
GST-rich^Cho (139)	11	6	6	9	1	25	12	51
Adipohemocyte^Cho (360)	54	6	9	66	22	91	69	49

Subcluster gene lists from Cho et al. mapped onto scRNA-Seq data from this study

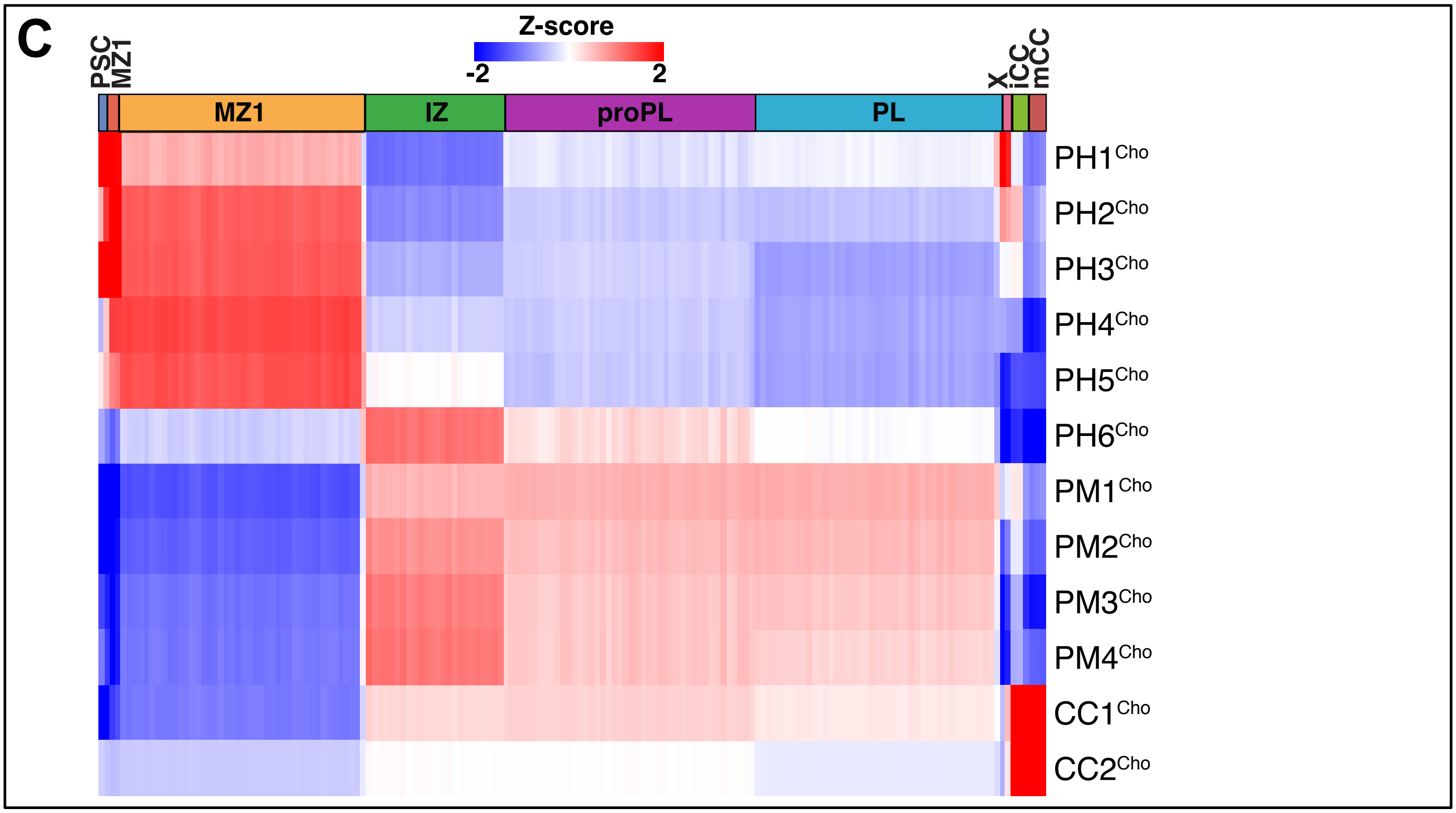

Comparison table of Sub-cluster gene lists from Cho et al. to this study

D highest number of genes in common / second highest number of genes in common

	PSC (368)	MZ1 (187)	MZ2 (97)	IZ (266)	proPL (73)	PL (973)	iCC (181)	mCC (104)	X (2525)
PH1^{Cho} (571)	60	24	3	7	1	41	15	12	235
PH2^{Cho} (347)	32	68	9	6	0	15	12	3	131
PH3^{Cho} (146)	38	54	11	1	0	2	9	4	31
PH4^{Cho} (91)	12	43	32	4	1	6	3	1	18
PH5^{Cho} (74)	20	23	32	11	0	6	5	5	3
PH6^{Cho} (297)	42	17	22	59	12	91	14	6	52
PM1^{Cho} (119)	6	1	1	52	38	88	36	10	33
PM2^{Cho} (221)	13	1	9	99	34	125	34	13	25
PM3^{Cho} (523)	47	7	13	116	31	234	35	15	92
PM4^{Cho} (637)	54	10	14	104	25	237	30	15	122
CC1^{Cho} (182)	7	18	3	24	17	35	73	50	22
CC2^{Cho} (236)	16	5	4	7	2	8	59	57	20

Comparison table of Sub-cluster cell percentages from Cho et al. to this study

E

This study	PSC	MZ1	MZ2	IZ	proPL	PL	X	iCC	mCC
% total cells	0.92%	1.32%	25.89%	14.82%	26.45%	26.07%	1.14%	1.52%	1.87%

Cho et al	PSC	PH1	PH2	PH3	PH4	PH5	PH6	PM1	PM2	PM3	PM4	CC1	CC2	GST-rich	Adipohemocyte
% total cells	0.98%	0.40%	0.42%	2.14%	27.19%	6.83%	2.33%	39.64%	9.37%	2.36%	3.00%	0.86%	0.58%	1.21%	0.92%

Supplementary File 2: Comparison of clusters from this study with clusters and subclusters identified by Cho et al. 2020.

Figure Page 1:
(A) To create a uniform platform for comparison of cell types identified in this study with the subcluster population described in Cho et al. 2020, we used AUCell to map enrichment of the gene lists from each of the primary cluster populations described in Cho et al. 2020 onto the cells within our identified clusters. Expressed as a heat map, the Cho et al. 2000 PSC cluster shows similar enrichment to the PSC cluster as defined in this study. For simplicity, we designate cell populations identified by the Cho et al. 2020, study with superscripted “Cho” and maintain our cluster names as we have described in the text. Thus PSC^{Cho} corresponds to PSC (described here). PH^{Cho} shows high expression in MZ1 and MZ2 cells, CC^{Cho} shows high expression in iCC and mCC cells, PM^{Cho} shows high expression in IZ, proPL, and PL cells. Adipohemocyte^{Cho} shows high expression in IZ, in a subset of proPL, and mCC cells. GST-rich^{Cho} shows high similarity to cluster X.
(B) The number of identical genes expressed in each population, as revealed by the two studies, are compared in this table. The clusters with the highest number of identical genes in common are highlighted. The analysis gives similar results to that seen by comparing AUCell scores, with the exception that Adipohemocyte^{Cho} shows highest overlap with PL, instead of IZ, proPL, or mCC.
(C) Heat map of subclusters identified by Cho et al. to our clusters as in **A**. AUCell of gene lists from Cho et al. subclusters usually map to more than one cluster from our study. Subclusters PH1-3^{Cho} show high expression in MZ1 cells, PH4-5^{Cho} show high expression in MZ2 cells, PH6^{Cho} shows high expression in IZ. PM1-4^{Cho} show high expression in IZ, proPL, and PL. CC1-2^{Cho} show high expression in iCC and mCC.

Figure Page 2:
(D) Similar comparisons as in **B**, except with subclusters. The comparisons are largely consistent with the trends seen in the AUCell analysis in **C**, except that PH1^{Cho} is more similar by gene comparison to X and PSC than PH1^{Cho} is similar to MZ1.
(E) Percentage of the total number of cells that is found in the subpopulations defined in the two studies. PSCs are of similar proportion. MZ1 is a little larger than PH1^{Cho} and PH2^{Cho} combined. MZ2 is comparable in size to PH4^{Cho}. IZ is larger than PH5^{Cho} and PH6^{Cho} combined. When added together, proPL and PL are of similar proportion to PM1-4^{Cho} combined. Cluster X is of similar size as GST-rich^{Cho}. iCC and mCC subpopulations taken together are slightly larger than the CC1^{Cho} and CC2^{Cho} (crystal cell numbers vary by age and environment).
